# Relationship between labor analgesia modalities and types of anesthetic techniques in categories 2 and 3 intrapartum cesarean deliveries

**DOI:** 10.17305/bb.2024.10186

**Published:** 2024-10-01

**Authors:** Tatjana Stopar Pintarič, Maja Pavlica, Mirjam Druškovič, Gorazd Kavšek, Ivan Verdenik, Polona Pečlin

**Affiliations:** 1 Department of Anaesthesiology and Intensive Therapy, University Medical Centre Ljubljana, Ljubljana, Slovenia; 2 Institute of Anatomy, Medical Faculty, University of Ljubljana, Ljubljana, Slovenia; 3 Department of Perinatology, Division of Obstetrics and Gynaecology, University Medical Centre Ljubljana, Ljubljana, Slovenia

**Keywords:** Emergency cesarean delivery, labor analgesia, remifentanil patient-controlled analgesia (remifentanil-PCA), epidural analgesia, regional anesthesia, general anesthesia, obstetric anesthesia, neonatal outcome.

## Abstract

General anesthesia (GA) is typically recommended for category 1 emergency cesarean delivery (CD). For categories 2–4 emergencies, either regional or GA can be used. The factors influencing the choice of anesthetic technique in these categories remain poorly understood. We analyzed the association between the type of labor analgesia and subsequent anesthetic techniques employed for intrapartum categories 2 and 3 CD. In a prospective longitudinal cohort study, 300 women were consequently enrolled and categorized according to Lucas’s classification of CD urgency. The techniques of anesthesia (GA, spinal, and epidural anesthesia [EA]) employed for CD were analyzed with respect to labor analgesia methods (remifentanil patient-controlled analgesia [remifentanil-PCA], EA, and nitrous oxide [N_2_O]). EA was the most frequent analgesic option (43.8%), followed by remifentanil-PCA (20.7%) and N_2_O (5.1%), while 30.4% of parturient women received no analgesia. All anesthetic methods showed a significant relationship with analgesic modalities (*P* < 0.001). Remifentanil-PCA was associated with a higher incidence of GA. Contraindication to EA was the primary factor related to the transition from remifentanil-PCA to GA. Most parturients who received EA were successfully converted to EA. Spinal anesthesia was the most common technique in women using N_2_O and those without labor analgesia. GA was associated with lower 5-min Apgar scores. The method of labor analgesia was associated with the anesthesia technique employed for categories 2 and 3 CD. This finding may guide patient counseling and intrapartum anesthetic planning. However, the analysis should be cautiously interpreted as the selection of anesthesia is a complex decision influenced by several clinical considerations.

## Introduction

Data collected from 169 countries, representing 98.4% of all global births, reveals that in 2015, an estimated 29.7 million deliveries (21.1%) were performed through cesarean delivery (CD) [1]. This proportion is expected to increase to 28.5% by the year 2030 [2]. Approximately 60% of women require anesthetic intervention during labor [3], and the appropriate anesthetic technique for CD is highly dependent on its urgency. The most widely used classification system for CD urgency is the four-scale category established by Lucas et al., which uses clinical criteria to determine the level of urgency based on potential maternal and/or fetal complications and whether it poses a life-threatening situation [4, 5].

The National Institute for Health and Clinical Excellence (NICE) guidelines recommend performing unplanned category 1 and 2 CDs quickly after making the decision. They suggest using a decision-to-delivery interval of 30 min for category 1 CDs and 30–75 min for category 2 CDs as an audit metric for obstetric units [6, 7]. However, there is ongoing debate about objective time limits for decision-to-delivery intervals, and no robust evidence exists linking this interval to outcomes [5]. For category 1 or “crash” CD, it is recommended that the time taken to achieve surgical anesthesia should be kept as short as possible. Unless there are contraindications, rapid sequence induction of general anesthesia is typically preferred for category 1 CDs because it consistently results in a shorter decision-to-delivery interval compared to spinal anesthesia [8, 9].

Existing literature on the anesthetic management of emergency CD focuses mainly on category 1 CD or emergency CD as a whole failing to address the unique challenges posed by categories 2 and 3 emergencies. Although regional anesthesia is generally recommended for non-crash CD, as it offers several benefits over general anesthesia, the choice of anesthetic modality is often not as straightforward as in category 1 CD [10]. This is particularly true regarding categories 2 and 3 CD, where general anesthesia may be considered in addition to spinal or epidural anesthesia, with the predictors of optimal anesthetic modality often poorly defined or understood [5, 11]. In both immediate- and intermediate-urgency intrapartum CD, maternal and fetal outcomes hinge on the obstetric anesthetist’s vigilance and coordinated effort due to time constraints and heightened risk, underscoring the critical significance of selecting the appropriate anesthetic technique [12]. While several anesthetic and obstetric factors and considerations may influence the choice of anesthetic technique in categories 2 and 3 CD, the association between the applied labor analgesic modality and the selection of subsequent cesarean anesthetic options has been scarcely investigated.

Several studies have shown that the choice of anesthetic technique used for cesarean delivery can impact the newborn’s outcome [13–15]. General anesthesia is usually associated with lower Apgar scores at 1 and 5 mins, while umbilical cord artery pH values do not differ. In general, all types of anesthesia appear to be safe, but regional techniques provide certain advantages for the well-being of newborns.

In cases where CD is required for a parturient with an existing labor epidural, it is customary to convert or “top-up” the epidural catheter by administering a more concentrated local anesthetic (LA) solution, typically in conjunction with a lipid-soluble opioid, to achieve surgical anesthesia [16]. Since existing considerations of the conversion of labor analgesia to anesthesia have primarily focused on neuraxial analgesia techniques, very little is known regarding the principles and practice associated with the effective and safe transition from other non-neuraxial labor analgesia modalities (e.g., nitrous oxide and intravenous opioids) to surgical anesthesia.

In parturients with remifentanil intravenous-patient controlled analgesia (remifentanil-PCA) who require CD, the selection of anesthetic technique for emergency CD is frequently unpredictable due to various factors, including individual patient preferences, obstetric considerations, potential contraindications to specific techniques, and the presence of labor pain, which can complicate the performance of neuraxial anesthesia. No previous studies have specifically examined the anesthetic and obstetric implications of remifentanil-PCA or other non-neuraxial analgesic methods on the subsequent approach to anesthesia for intrapartum CD. To facilitate informed counseling regarding the relationship between labor analgesia and subsequent anesthetic choices in emergency CD, we aimed to analyze the anesthesia techniques employed in categories 2 and 3 emergency CD with respect to different modalities of labor analgesia used in labors in our delivery unit.

## Materials and methods

### Study design and patient selection

The research was conducted at the labor and delivery unit of the Perinatology Department, Division of Obstetrics and Gynaecology, at the University Medical Centre Ljubljana. This unit handles approximately 5000 deliveries annually, featuring a 70% utilization rate of neuraxial and remifentanil analgesia with a 1:1 ratio and a CD rate of 21%. The study included women who underwent categories 2 and 3 emergency CD between March and October 2021. We excluded cases of category 1 CD (obligatory general anesthesia) and category 4 CD (without prior labor analgesia). Women with planned (“elective”) CD were also excluded from the analysis. A CD was classified as “elective” if it was planned in advance, allowing for careful preparation and scheduling, rather than waiting for labor to begin naturally, or “emergency” if it was performed after the onset of labor or in response to a sudden medical condition that makes a vaginal delivery risky for the mother or baby.

Per institutional standard operative protocol, crash CD (category 1) is performed under general anesthesia in the absence of known absolute contraindication, whereas regional anesthesia is recommended for cases classified under urgency levels 2–4.

### Categorization into urgency groups

The indication for the intrapartum CD was carefully documented at the time of the decision, and the classifications adhered to the urgency-level categorization system proposed by Lucas et al. [4]. The proposed indications for the categorization of patients into specific groups are as follows.

*Group 1 (“crash” CD: An immediate threat to maternal or fetal life):* Severe pathological cardiotocography (CTG) readings, such as sustained fetal bradycardia; massive hemorrhage from placenta previa or abruption accompanied by hemodynamic instability or pathological CTG patterns; uterine rupture; failed extraction of the second twin due to complications like a neglected transverse position; maternal cardiac arrest; and instances of failed instrumental delivery with evidence of severe fetal distress.

*Group 2 (Maternal or fetal compromise not immediately life-threatening)*: Failed instrumental delivery due to prolonged second stage of labor, fetal distress, as indicated by non-life-threatening pathological CTG or a fetal scalp pH measurement below 7.20; fetal malposition during advanced labor; and placental abruption cases with mild bleeding and fetal compromise.

*Group 3 (Need for early delivery without maternal or fetal compromise):* Bleeding placenta previa without concurrent hemodynamic instability; cephalo-pelvic disproportion (CPD), poor progress of labor; and cases of pathological CTG mandating completion of delivery in approximately 60 min; and fetal malposition at the outset of labor.

*Group 4 (Delivery at a suitable time for the patient and maternity team):* Failed induction, planned cesarean delivery with initial signs of labor; medical indications necessitating CD due to maternal conditions requiring stabilization, such as preeclampsia, appendicitis, or pancreatitis in the third trimester or fetal conditions such as intrauterine growth restriction.

### Labor analgesia methods

The choice of labor analgesia method was made by women during labor, following consultations with anesthesiologists, obstetricians, and midwives. The available modalities in our obstetric unit include remifentanil-PCA, epidural analgesia, nitrous oxide, and pethidine. All participants had the same analgesic options available to them.

#### Remifentanil patient-controlled analgesia (PCA)

In our institution, labor epidural and remifentanil-PCA are equally represented as labor analgesic choices. Since its introduction in 2013, remifentanil-PCA has been widely used for medical, obstetrical, and patient-driven indications, with over 1300 cases of administration annually. Remifentanil-PCA administration adhered to the procedural guidelines of the Department of Anaesthesia and Intensive Therapy at the University Medical Centre Ljubljana. Remifentanil hydrochloride (Ultiva, GlaxoSmithKline, Oslo, Norway) was prepared in saline at a concentration of 40 µg mL^−1^. The administration followed a stepwise approach, commencing at 20 µg and escalating to a maximum of 40 µg, with a bolus duration of 20 s and a lockout interval of 2 min, devoid of any background infusion. The anesthesiology staff adjusted the dose based on the patient’s request. Bolus dose increase occurred if pain intensity (assessed by an 11-point numerical rating scale) rose and the patient’s respiratory rate was > 9 breaths min^−1^, with SpO_2_ ≥ 94%, heart rate > 50 min^−1^, and sedation score ≤ 2 on a five-point scale. Throughout the procedure, women received dedicated one-to-one midwifery care and underwent continuous monitoring utilizing a Capnostream^®^ capnograph (Oridion^®^, Jerusalem, Israel) equipped with an oral–nasal cannula for sampling from the nose and mouth (Oridion^®^). All patients received supplemental oxygen at a rate of 2 L per minute through a nasal catheter. The respiratory monitor continuously recorded waveform data for end-tidal CO_2_, respiratory rate, SpO_2_, and heart rate. Alarms were triggered in cases of oxygen desaturation (SpO_2_ < 94%), bradypnea (respiratory rate < 8 min^−1^), or apnoea exceeding 20 s. Staged interventions were initiated, commencing with verbal instruction to take a deep breath or a gentle tap if no response was elicited. Continuous fetal heart rate monitoring was performed with the Hewlett Packard Viridia Series 50IP^®^ or Philips 50XM^®^ CTG equipment. Remifentanil administration was halted in the event of pathological CTG changes, including reduced variability, bradycardia, tachycardia, or late decelerations. According to the institutional guidelines, contraindications for remifentanil-PCA use in labor include patient refusal, documented history of allergy to opioids, parenteral opioid medication administered within the previous two hours, opioid drug abuse, obstructive sleep apnoea, or unavailability of 1:1 midwife care [17–19].

#### Epidural analgesia

Epidural analgesia has been consistently administered around the clock in our institution since 2013, adhering to the established procedural guidelines of the Department of Anaesthesia and Intensive Therapy at the University Medical Centre Ljubljana. Catheter insertion was conducted with patients in a seated position. The epidural space was identified using an 18-gauge Tuohy needle (PORTEX^®^ CSE cure^®^ Combined Spinal Epidural System, Smiths Medical, Minnesota, USA) inserted in the midline, employing the loss of resistance technique with air or saline at the L3–L4 or L4–L5 intervertebral space. Subsequently, a 20-gauge multihole catheter was inserted. A test bolus of 3 mL of lidocaine 2% was administered to assess epidural placement, followed by an initial 20 mL infusion of a mixture containing 0.1% bupivacaine and 2 µg mL^−1^ fentanyl. The epidural analgesia was delivered using the same LA mixture via a combination of programmed intermittent epidural bolus (PIEB) and patient-controlled epidural analgesia (PCEA) techniques using a Rhythmic™ Evolution pump (Micrel Medical Devices, Athens, Greece). As per the institutional protocol, contraindications for EA encompass patient refusal, localized sepsis at the puncture site, thrombocytopenia, coagulopathy or anticoagulant therapy, signs of cardiovascular instability, and significant lumbar deformity or prior major spinal surgery.

#### Nitrous oxide

Nitrous oxide (N_2_O) was provided by midwives using an intermittent approach of 50%–70% N_2_O in oxygen administered via a handheld facemask or mouthpiece. The parturient initiates inhalation, generating the flow of N_2_O. The peak effect is achieved within 30–40 s, after which the parturient is instructed to exhale into the mask or mouthpiece to ensure a complete elimination of respired particles through the system.

#### Pethidine

Per institutional protocol, eligible patients who opted for this modality received 50–100 mg intravenous pethidine injection. However, pethidine was used in only three parturients who were excluded from further analysis.

### Anesthetic techniques

#### General anesthesia

General anesthesia was provided via rapid sequence induction. Prior to induction, a pre-oxygenation regimen consisting of 4–5 vital-capacity breaths of pure oxygen was administered, followed by intravenous injection of 5 mg/kg of thiopental or 2 mg/kg of propofol and 1 mg/kg of succinylcholine chloride or rocuronium (1 mg/kg). Subsequently, endotracheal intubation was performed, and anesthesia was maintained with sevoflurane in a 60/40 nitrous oxide/oxygen mixture. For patients initially given suxamethonium, 0.5 mg/kg of rocuronium was administered for maintenance of neuromuscular blockade. After cord clamping, fentanyl 3–5 mg/kg was added intravenously.

#### Spinal anesthesia

Spinal anesthesia was administered through a 25-gauge Sprotte needle, inserted in the midline and positioned at the L3–L4 intervertebral space with the injection of 7.5–11 mg of bupivacaine and 25 µg of fentanyl. Subsequently, the patient was positioned in a supine posture with a 15∘ left lateral tilt, and a 15∘ Trendelenburg position was adopted to enhance the cephalic distribution of the anesthetic agents. We considered anesthesia adequate if it resulted in an upper sensory block extending to the T4 level.

#### Epidural anesthesia

In patients with existing epidural analgesia regimens, epidural anesthesia for emergency intrapartum CD was established with an epidural top-up of 2% lidocaine to a total of 20 mL, commencing in the delivery room. We considered anesthesia adequate if it resulted in an upper sensory block extending to the T4 level.

### Ethical statement

The study was approved by the National Medical Ethics Committee of the Republic of Slovenia (approval No: 0120-219/2021/3). Written informed consent was obtained from all study participants prior to their enrollment.

### Statistical analysis

The following data were recorded for statistical analyses: demographic and obstetric data of parturients (age, body mass index (BMI), parity, and CD history); the urgency level of CD graded from categories 1–4; the type of labor analgesia employed (whether none, N_2_O, epidural, or remifentanil-PCA); the method of anesthesia administered (such as general anesthesia, spinal anesthesia, or epidural anesthesia); and the rate of conversions from regional anesthesia to general anesthesia. Additionally, we also recorded adverse neonatal outcomes, including mortality, Apgar scores below 7 at 5 min, umbilical cord artery pH levels below 7.0, and base excess values below 12. Descriptive statistics for parametric data are expressed as mean ± standard deviation (SD). The Student *t*-test and chi-square test were employed for comparing continuous and categorical variables, respectively. A significance threshold was set at *P* < 0.05. All statistical analyses were performed with IBM SPSS Statistics for Windows Version 27.0 (Armonk, NY, USA: IBM Corp).

## Results

**Figure 1.**
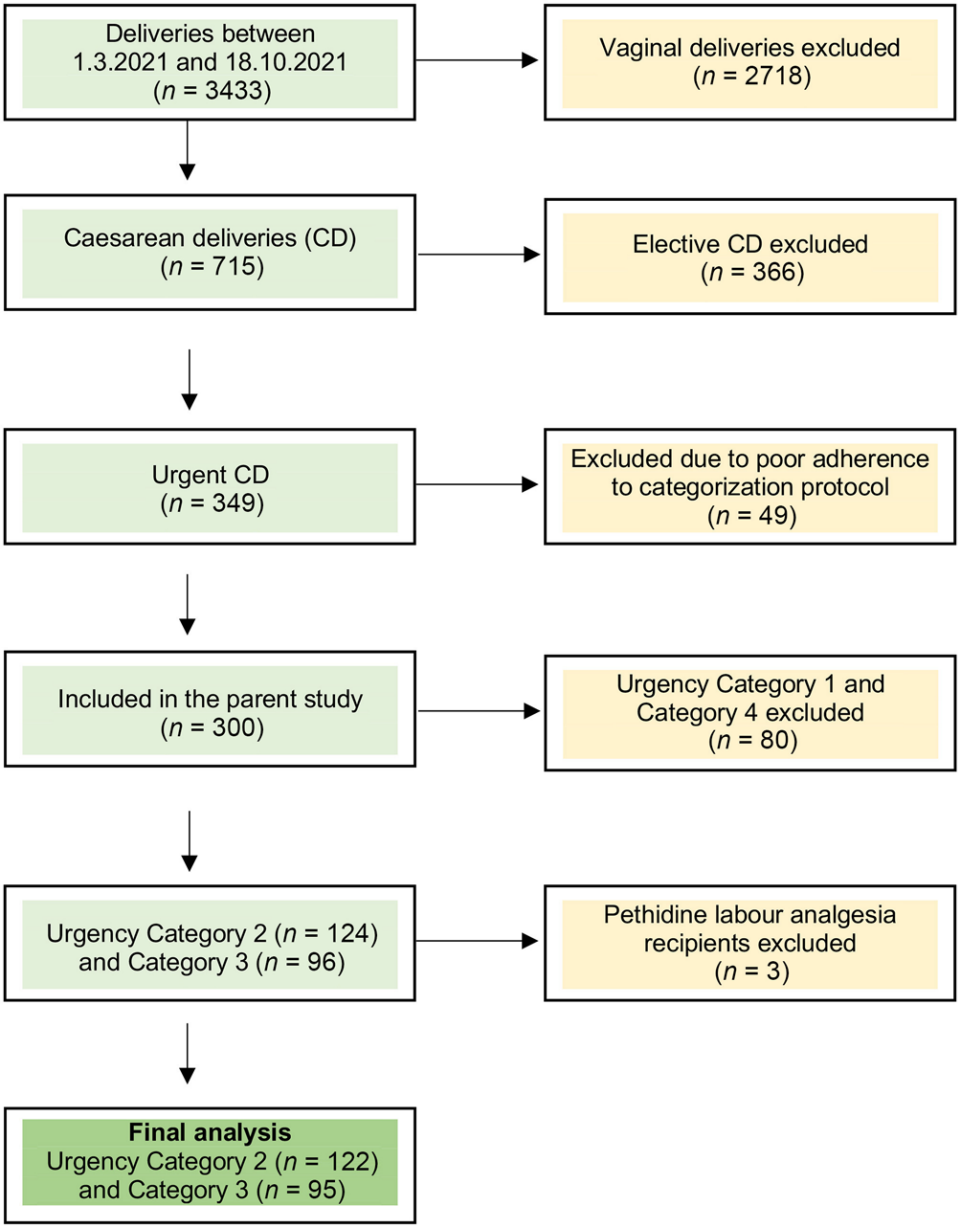
**Flowchart of the patient enrolment process.** CD: Caesarean delivery.

A total of 300 women were prospectively enrolled in this study, with the distribution across urgency categories depicted in Table 1. Excluded from the analysis were women falling under category 1 CD, where timely delivery is imperative due to predominantly irreversible causes of fetal or maternal distress, such as umbilical cord prolapse, uterine scar dehiscence, and placental abruption. Our center utilizes general anesthesia for category 1 CD to minimize the time from decision to delivery as much as possible. Women in category 4 CD were excluded because they had not received prior labor analgesia. During the study period, 49 potentially eligible CDs were excluded from the analysis due to non-compliance with the study protocols. Additionally, data for two women were missing, and three cases involving pethidine labor analgesia were excluded from the final analysis. Of the remaining participants, 122 (41.3%) were categorized under level 2 CD, while 95 (32%) were categorized as level 3 CD, forming the basis of our final analytical cohort. Figure 1 illustrates the patient screening and selection process.

**Table 1:** Distribution of parturients according to the level of urgency of intrapartum caesarean delivery

Level of urgency*	**Interpretation**	***n* (%)**
1	Immediate threat to maternal/fetal life	50 (16.7%)
2	Maternal or fetal compromise that is not immediately life-threatening	124 (41.3%)
3	Need for early delivery without maternal or fetal compromise	96 (32.0%)
4	Delivery at a suitable time for the patient and maternity team	27 (9.0%)

Note: *Per the categorization of urgency of cesarean delivery proposed by Lucas et al. [4]. The green highlight indicates the categories of interest in the present study.

The main indications for category 2 CD were fetal distress from non-immediately threatening pathological CTG (53 cases, 43%), fetal malposition during advanced labor (18 cases, 15%), CPD (17 cases, 14%), and poor progress of labor (12 cases, 10%). The commonest indications for category 3 CD included poor progress of labor (39.6%), CPD (27.1%), and previously planned cesarean delivery with initial signs of labor (12.5%).

Table 2 presents a comprehensive overview of the demographic, obstetric, and anesthetic data for participants in categories 2 and 3 CD. These data are stratified based on the type of labor analgesia administered, namely, no analgesia, N_2_O, epidural analgesia, and remifentanil-PCA. Epidural analgesia was the most frequent analgesic choice in this cohort (43.8%), followed by remifentanil-PCA (20.7%) and N_2_O (5.1%), while about a third of the parturients (30.4%) required no labor analgesia. We observed no statistically significant differences in age, BMI, or previous CD history among the various labor analgesia groups. However, significant differences were noted in parity. Epidural analgesia was the most commonly used labor analgesic method among nulliparous women. The remifentanil-PCA group had the highest proportion of parturients with previous CD history. Among multiparous women without previous CD, the highest proportion was found in the no-analgesia group, followed by the remifentanil-PCA group.

**Table 2:** Maternal demographic and obstetric data with respect to different labor analgesic methods

	**No analgesia**	**Nitric oxide**	**Epidural analgesia**	**Remifentanil-PCA**	**Total**	***P* value**
Number	66 (30.4%)	11 (5.1%)	95 (43.8%)	45 (20.7%)	217	
Age	32.6 ± 5.9	31.3 ± 4.9	31.5 ± 4.6	32.0 ± 5.6	31.9 ± 5.2	ns (0.641)
BMI (kg/m^2^)	24.5 ± 5.2	22.3 ± 3.6	24.7 ± 3.9	26.2 ± 5.5	24.8 ± 4.8	ns (0.060)
Nulliparous	33/66 (50.0%)	6/11 (54.0%)	93/95 (98.0%)	24/45 (53.3%)	156 (70.7%)	< 0.001
Multiparous with previous CD	15/33 (45.5%)	3/5 (60%)	1/2 (50%)	11/21 (52%)	30/61 (49.0%)	ns (0.918)
Multiparous without previous CD	18/33 (54.5%)	2/5 (40.0%)	1/2 (50.0%)	10/21 (48.0%)	31/61 (51.0%)	ns

Data are presented as mean (SD) or *n* (%) and with a significance threshold at *P* < 0.05. CD: Caesarean delivery; Remifentanil-PCA: Remifentanil intravenous-patient controlled analgesia; SD: Standard deviation; ns: Not significant.

**Table 3:** Selection of anesthetic techniques with respect to labor analgesic modalities

	**No analgesia**	**N_2_O**	**EA**	**Remifentanil-PCA**	***P* value**
General anesthesia	31 (47%)	4 (36.4%)	28 (29.5%)	27 (60%)	<0.001
Epidural anesthesia	0	0	66 (69.5)	0	
Spinal anesthesia	35 (53%)	7 (63.6%)	1 (1.0%)	18 (40%)	
Conversion from epidural anesthesia to general anesthesia	0	0	3 (3.3%)	0	

*P* is significant at < 0.05. EA: Epidural analgesia; Remifentanil-PCA: Remifentanil intravenous-patient controlled analgesia; GA: General anesthesia; N_2_O: Nitrous oxide.

Table 3 summarizes the findings regarding the association of anesthetic techniques with different analgesic modalities in category 2 and 3 CD. All anesthetic methods showed a statistically significant relationship to analgesic modalities (*P* < 0.001). 69.5% of parturients who received epidural analgesia subsequently required conversion to epidural anesthesia for CD. Parturients utilizing other analgesic methods did not require epidural anesthesia. 53% of parturients who received no labor analgesia and 63.6% of those who received N_2_O transitioned to spinal anesthesia, while the rest had general anesthesia. On the other hand, 60% of parturients who received labor remifentanil-PCA transitioned to general anesthesia for intrapartum CD, while the rest utilized spinal anesthesia. Further analysis of the selection of anesthetic technique with respect to indications for remifentanil-PCA showed that among parturients receiving remifentanil-PCA, “contraindication to epidural analgesia” due to patient refusal was significantly associated with the use of general anesthesia for CD (Table 4).

**Table 4:** Mode of anesthetic transition with respect to indications for remifentanil-PCA

	**GA**	**RA**	***P* value**
Contraindications for EA	6	0	0.04
Failed EA	2	0	ns
Obesity (BMI > 35 kg/m^2^)	3	0	ns
Previous CD	5	10	ns
Twin and breech vaginal deliveries	2	1	ns
Others (patient preferences)	9	7	ns

*P* is significant at < 0.05. BMI: Body mass index; CD: Caeserean delivery; EA: Epidural analgesia; GA: General anesthesia; RA: Regional anesthesia; Remifentanil-PCA: Remifentanil intravenous-patient controlled analgesia; ns: Not significant.

The association between neonatal outcome data and the different analgesic modalities and subsequent anesthetic techniques is presented in Table 5. No differences in Apgar scores were observed between analgesic modalities when converted to neuraxial anesthesia. However, in those converted to GA, significantly lower Apgar scores were observed only in the no-analgesia group (*P* ═ 0.002). This finding appears to be associated with an elevated incidence of prematurity within this particular group. Upon further analysis of the subgroup with no analgesia in cases of prematurity, it was determined that most cases necessitated CD due to fetal malposition or fetal distress subsequent to the initiation of premature labor. In such cases, intrapartum analgesia was deemed unnecessary. Other neonatal parameters, including pH and base excess, showed no statistically significant relationship with the analgesic groups.

**Table 5:** Relationship between neonatal outcome data and analgesic/anesthetic modalities

	**No analgesia**	**N_2_O**	**EA**	**Remifentanil-PCA**	***P* value**
5-min Apgar < 7 after RA (spinal + EA)	1/35 (2.9%)	0/7	1/70 (1.4%)	0	ns
5-min Apgar < 7 after GA	5/31 (16.1%)	0/4	0/25	0/27	0.022
pH umbilical artery < 7 after RA (spinal + EA)	0	0	0	0	ns
pH umbilical artery < 7 after GA	0	0	0	0	ns
Base excess umbilical artery < −12 after RA	1/35 (2.9%)	0	3/70 (4.3%)	0	ns
Base excess umbilical artery < –12 after GA	2/31 (6.5%)	0	0	0	ns
Preterm birth < 37 weeks, *n* (%)	38 (66%)	2 (18%)	0 (0%)	2 (0.4%)	< 0.001

*P* is significant at < 0.05. NICU: Neonatal intensive care unit; EA: Epidural analgesia; GA: General anesthesia; RA: Regional anesthesia; Remifentanil-PCA: Remifentanil intravenous-patient controlled analgesia; N_2_O: Nitrous oxide; ns: Not significant.

## Discussion

This study investigated the relationship between different methods of labor analgesia (neuraxial, remifentanil-PCA, and N_2_O) and the type of anesthesia technique (general, spinal, or epidural) employed for intrapartum CD of categories 2 and 3 urgencies. All anesthetic techniques were found to have a statistically significant relationship with the type of labor analgesia priorly administered. The majority of women who received epidural analgesia transitioned to epidural anesthesia. Conversely, among women who received remifentanil-PCA for labor analgesia, 60% transitioned to general anesthesia, with the remaining 40% undergoing spinal anesthesia. Patient refusal of epidural analgesia was the primary factor associated with the transition to general anesthesia in the remifentanil-PCA group. More than half of the women who received no labor analgesia and those who received N_2_O transitioned to spinal anesthesia. Overall, the analysis suggests that the modality of labor analgesia may be an important predictor of the subsequent anesthetic approach in the event of CD of intermediate urgency. The specific findings may be useful for patient counseling and can inform and guide anesthetic and obstetric preparedness in intrapartum CD.

Currently, various pain relief options are accessible for labor, including neuraxial analgesia (e.g., epidural), parenteral opioids, and inhalational analgesia. The choice of labor pain management in women is shaped by factors, such as health, demographics, and attitudes, with varying impacts depending on the specific technique employed [20]. Guidelines from both the American Society of Anaesthesiologists (ASA) and the American College of Obstetricians and Gynaecologists (ACOG) recommend epidural analgesia as the most adaptable, efficient, and least neurologically depressive analgesic method in obstetrics [21], with new techniques like PIEB, dural puncture epidural, and ultrasound-guided neuraxial approaches offering improved precision [22]. Although neuraxial analgesia offers highly effective labor pain relief, its utilization in labor can be influenced by factors like availability, contraindications, and individual preferences [23]. Severe conditions like deep vein thrombosis, pulmonary embolism, mechanical heart valve, arrhythmia, severe scoliosis, or post-surgical spine instrumentation may preclude optimal and timely administration of neuraxial labor analgesia [24]. Other analgesic strategies are indicated when central neuraxial analgesia is contraindicated, technically infeasible, or if the patient’s preference dictates.

Epidural analgesia was the most frequently administered analgesic option in the analyzed cohort, reflecting current clinical trends and recommendations. While epidural analgesia does not inherently elevate the risk of CD, approximately 10% of parturients utilizing epidural analgesia during labor may require emergency CD [25, 26]. To enable surgical anesthesia in parturients with an existing labor epidural, it is standard practice to convert or “top-up” the epidural catheter by administering a more concentrated LA solution, often combined with a lipid-soluble opioid when a CD is indicated. It was shown that lidocaine 2%, with or without fentanyl, provides the quickest onset of sensory blockade during conversion, and the inclusion of ropivacaine 0.75% in the epidural top-up solution diminishes the necessity for additional supplementation in surgery [16]. Efficient conversion of labor analgesia to surgical anesthesia may serve as a valuable quality of care indicator in addition to affirming the prior efficacy of the labor analgesia modality [16].

The observation that epidural anesthesia was solely associated with epidural analgesia suggests that this anesthetic technique is primarily favored in the context of priorly established neuraxial access with epidural analgesia. Accordingly, patients with labor epidural analgesia should be counseled about the overall likelihood of transitioning to anesthesia via top-up of existing analgesia and that in the event of failed conversion of epidural analgesia to surgical anesthesia, general but not spinal anesthesia is typically likely. It was reported that the likelihood of unsuccessful conversion from labor epidural analgesia to anesthesia rises with higher bolus administrations in labor, heightened urgency for CD, and care administered by a non-obstetric anesthesiologist [27]. Our analysis also showed a low conversion rate from epidural to general anesthesia. The Royal College of Anesthetists guidelines recommend that the conversion rate from neuraxial to general anesthesia for category 1 and categories 1–3 CD should be maintained at less than 15% and 5%, respectively [28]. Although we observed no association between spinal anesthesia with previous epidural analgesia, a retrospective analysis of parturients who received epidural labor analgesia but needed subsequent CD under regional anesthesia found that spinal anesthesia resulted in reduced time from anesthesia to surgical incision and total anesthetic time, lower postoperative pain scores, and decreased morphine dosage when compared to epidural anesthesia [26]. Similarly, rapid sequence spinal anesthesia is increasingly preferred over general anesthesia for many category 1 CD indications [11]. Nonetheless, administering spinal anesthesia after epidural analgesia can lead to an unanticipated profound blockade, potentially reaching total sensory and motor spinal anesthesia [29, 30].

Our analysis has shown that remifentanil-PCA is associated with a higher incidence of general anesthesia in categories 2 and 3 emergency CD. Several factors potentially influence this observed association. Firstly, women opting for remifentanil-PCA often present with contraindications for epidural analgesia. Additionally, certain obstetric conditions, such as a history of previous CD, twin gestation, or a breech presentation, may pose heightened risks with epidural analgesia, prompting a preference for alternative analgesic approaches [31–34]. A recent study by Parissenti et al. [35] demonstrated that EA constitutes a significant risk factor for the failure of vaginal breech delivery, leading to an increased likelihood of intrapartum CD. Jaschevatzky et al. [33] reported higher rates of operative vaginal deliveries and higher pre-term perinatal mortality in twin deliveries with EA, despite similar neonatal status (as assessed by the Apgar score at one minute) in both the EA and control groups. Similarly, in a case series of parturients with multiple gestations who delivered vaginally, a higher incidence of low Apgar-minus-color scores at 1 min among the second twins of at least 36 weeks gestation was reported in the EA group [32]. Our analysis of the selection of anesthetic technique with respect to indications for remifentanil-PCA shows that EA refusal was the only statistically significant factor related to the increased transition to general anesthesia for CD. Given the higher likelihood of encountering general anesthesia in laboring women who received remifentanil-PCA, it is imperative that parturients are counseled beforehand regarding the potential need for transition to general anesthesia and the associated complications thereof (especially a higher likelihood of gastric paresis with the consequent risk of regurgitation and aspiration) [36], should intrapartum CD become necessary. Despite many controversies regarding remifentanil efficacy and safety, remifentanil-PCA shows comparable delivery and neonatal outcomes to epidural analgesia within any of the Ten Groups Classification System (TGCD) labor types [17, 19]. In the present analysis, remifentanil-PCA and epidural anesthesia also demonstrated comparable neonatal outcomes. As previously shown in other studies [13–15], general anesthesia typically correlated with a higher incidence of neonates having low Apgar scores at the fifth minute, with no significant difference in umbilical cord artery pH and base excess values.

Furthermore, our analysis shows that women who received no labor analgesia and those who received N_2_O mainly transitioned to spinal anesthesia, suggesting that this anesthetic option is associated with parturients who are naïve to invasive analgesic modalities. A retrospective analysis found that only a small proportion of laboring women (mostly nulliparous women with an initial preference for non-medical birth) chose nitrous oxide for analgesia during labor and delivery, with the majority ultimately converting to neuraxial analgesia, suggesting the minimal analgesic effect of nitrous oxide and the need to counsel parturients on the potential of analgesia conversion [37]. Nevertheless, women using N_2_O experience enhanced maternal satisfaction and coping compared to those without analgesia, despite its lower analgesic effectiveness compared to neuraxial labor analgesia [38], suggesting that pain relief is not the only contributor to maternal satisfaction with labor analgesia [39].

Our cohort demonstrated no adverse neonatal outcomes attributable to hypoxia. Neonatal parameters (including pH and base excess) revealed no statistically significant differences among groups. Furthermore, Apgar scores showed no variation between analgesic modalities when transitioning to neuraxial anesthesia. Conversely, the conversion to GA was associated with significantly lower Apgar scores within the no-analgesia group (*P* ═ 0.002). Further analysis revealed a significantly higher incidence of preterm birth (before the 37th week of gestation) in the no-analgesia group (66%) compared to those receiving EA or remifentanil-PCA (<1%). In the no-analgesia group, most CDs were performed due to abnormal fetal positioning early in labor, often involving presentations, such as breech, footling, or transverse lie. These laboring mothers had not yet requested intrapartum analgesia. The disparity in the gestational age notably affected the Apgar score assessment of the newborn, especially when using general anesthesia to perform CD. The effects of general anesthesia on the newborn are multifactorial and can vary depending on the specific circumstances of the CD and the individual responses of both the mother and the baby. General anesthesia medications readily cross the placenta, potentially causing neonatal central nervous system depression that manifests as respiratory difficulties and may lead to lower Apgar scores [40].

Although the results of our analysis indicate that the type of labor analgesia used could be a significant factor in predicting the subsequent anesthetic approach for intrapartum CD, it is crucial to interpret our findings with caution. First, the study’s observational, non-randomized design inherently limits causal inference due to the potential for uncontrolled confounding variables. Variations in patient characteristics or clinical practices across groups may bias the observed results. Nevertheless, as randomized controlled trials (RCTs) on the effects of labor analgesia and anesthetic choice in categories 2 and 3 emergency CD are unlikely in the near future, patient counseling regarding potential associations between anesthetic methods in these scenarios will continue to rely primarily on observational data. Second, our analysis has primarily examined the association between the chosen analgesic modality and the subsequent anesthetic transition. We did not consider the specific indications and rationale guiding the labor analgesic/anesthetic choice. The choice of analgesic technique during labor and subsequent anesthetic management for intrapartum cesarean delivery (CD) is a complex decision influenced by diverse factors. These include patient preferences (e.g., prior experience, pain perception, concerns about nerve damage), anesthesia-related considerations (e.g., indications and contraindications), obstetric circumstances, fetal well-being, and other clinical factors [41]. Thus, it is imperative to recognize that the choice of anesthesia is not solely a consequence of preexisting labor analgesia but rather a complex and carefully considered decision aimed at optimizing the outcome for both the parturient and the neonate. Third, as a single-center study, the generalizability of our results to a broader population may be limited. However, our position as Slovenia’s largest tertiary center, handling one-third of the nation’s births and diverse pregnancy referrals, suggests that our findings may have relevance for other major obstetric centers. While a larger, multicenter retrospective study could offer more definitive insights, our study provides valuable initial data on this important topic.

## Conclusion

This prospective cohort study investigated the relationship between various labor analgesia methods (neuraxial, remifentanil-PCA, and N_2_O) and the anesthesia technique (general, spinal, or epidural) for intrapartum CD of categories 2 and 3 urgencies. Our analysis shows that more than half of women utilizing remifentanil-PCA for labor analgesia transitioned to general anesthesia for intrapartum CD, with a smaller proportion undergoing spinal anesthesia. Contraindications to epidural analgesia were significantly associated with transitioning from remifentanil-PCA to general anesthesia. On the other hand, most women who received epidural analgesia continued with epidural anesthesia, while those in other analgesic groups seldom underwent this approach. Additionally, more than half of women who received no labor analgesia or N_2_O underwent spinal anesthesia, suggesting a preference for this option among parturients without prior invasive analgesia. Our observations may be useful for counseling patients and guiding anesthetic preparedness in categories 2 and 3 intrapartum CD. Nevertheless, it is imperative to approach these findings with caution, as our analysis primarily explored the association between the types of analgesic modality and subsequent anesthetic transitions without consideration of specific factors that impact the selection of anesthetic techniques. The decision regarding anesthesia technique during intrapartum CD is complex, influenced by a spectrum of factors extending beyond preexisting labor analgesia.

## Data Availability

The data reported in this study are available upon reasonable request to the corresponding author.
